# Gut Microbiota Is a Major Contributor to Adiposity in Pigs

**DOI:** 10.3389/fmicb.2018.03045

**Published:** 2018-12-10

**Authors:** Hua Yang, Yun Xiang, Kelsy Robinson, Junjun Wang, Guolong Zhang, Jiangchao Zhao, Yingping Xiao

**Affiliations:** ^1^Institute of Quality and Standards for Agro-Products, Zhejiang Academy of Agricultural Sciences, Hangzhou, China; ^2^Institute of Animal Husbandry and Veterinary Medicine, Jinhua Academy of Agricultural Sciences, Jinhua, China; ^3^Department of Animal and Food Sciences, Oklahoma State University, Stillwater, OK, United States; ^4^Beijing Advanced Innovation Center for Food Nutrition and Human Health and State Key Laboratory of Animal Nutrition, China Agricultural University, Beijing, China; ^5^Department of Animal Science, Division of Agriculture, University of Arkansas, Fayetteville, AR, United States

**Keywords:** microbiota, fecal microbiota transplantation, fat metabolism, adipogenesis, obesity, pigs

## Abstract

Different breeds of pigs vary greatly in their propensity for adiposity. Gut microbiota is known to play an important role in modulating host physiology including fat metabolism. However, the relative contribution of gut microbiota to lipogenic characteristics of pigs remains elusive. In this study, we transplanted fecal microbiota of adult Jinhua and Landrace pigs, two breeds of pigs with distinct lipogenic phenotypes, to antibiotic-treated mice. Our results indicated that, 4 weeks after fecal transplantation, the mice receiving Jinhua pigs’ “obese” microbiota (JM) exhibited a different intestinal bacterial community structure from those receiving Landrace pigs’ “lean” microbiota (LM). Notably, an elevated ratio of Firmicutes to Bacteroidetes and a significant diminishment of *Akkermansia* were observed in JM mice relative to LM mice. Importantly, mouse recipients resembled their respective porcine donors in many of the lipogenic characteristics. Similar to Jinhua pig donors, JM mice had elevated lipid and triglyceride levels and the lipoprotein lipase activity in the liver. Enhanced expression of multiple key lipogenic genes and reduced angiopoietin-like 4 (*Angptl4*) mRNA expression were also observed in JM mice, relative to those in LM mice. These results collectively suggested that gut microbiota of Jinhua pigs is more capable of enhancing lipogenesis than that of Landrace pigs. Transferability of the lipogenic phenotype across species further indicated that gut microbiota plays a major role in contributing to adiposity in pigs. Manipulation of intestinal microbiota will, therefore, have a profound impact on altering host metabolism and adipogenesis, with an important implication in the treatment of human overweight and obesity.

## Introduction

Obesity is a global epidemic and a leading preventable cause of death worldwide (Srivastava and Apovian, 2018; Upadhyay et al., 2018). Different breeds of pigs have distinct genetic makeups that give rise to diverse phenotypic traits such as fat metabolism (Wu et al., 2013; Pajarillo et al., 2014). Landrace pigs are a widely adopted commercial breed that was developed originally in Denmark by crossing native pigs with the Large White swine, and are well known for their rapid growth and high lean carcass yield (Xiao et al., 2017a). In contrast, Jinhua pigs are a local Chinese breed in Zhejiang Province of China. It exhibits slow growth rate and high intramuscular fat (IMF) content (Wu et al., 2013). The obvious difference in the propensity for adipogenesis between Landrace and Jinghua pigs makes them excellent models to study the factors influencing adipogenesis and obesity (Wu et al., 2013).

A number of proteins are known to be involved in fat metabolism. Acetyl-CoA carboxylase (ACC) and fatty acid synthase (FAS) are two key enzymes involved in *de novo* fatty acid synthesis, while fatty acid binding protein 4 (FABP4) is critical in the uptake, transport, storage and metabolism of fatty acids (Strable and Ntambi, 2010; Lenhard, 2011). Two lipogenic transcriptional factors including sterol response element binding transcription factor 1 (SREBF1) and MLX-interacting protein like (MLXIPL) enhance lipoprotein lipase (LPL)-mediated triglyceride deposition into adipocytes by inducing the expression of *ACC* and *FAS* (Strable and Ntambi, 2010; Wang et al., 2015) and suppressing intestinal epithelial expression of angiopoietin-like 4 (*ANGPTL4*), a circulating LPL inhibitor involved in lipid metabolism and susceptible to regulation by gut microbiota (Backhed et al., 2004; Dijk and Kersten, 2016; Olshan and Rader, 2018). The *ANGPTL4* gene is under tight transcriptional control of peroxisome proliferator activated receptors (PPARα and PPARγ), which govern lipid metabolism in the liver and white adipose tissue, respectively (La Paglia et al., 2017). LPL is a well-known key regulator of fatty acid release from triglyceride-rich lipoproteins, leading to an increased cellular uptake of fatty acids and triglyceride accumulation in adipocytes (Preiss-Landl et al., 2002).

Similar to humans and other vertebrates, the gastrointestinal tract of pigs harbors trillions of commensal microorganisms, primarily bacteria, which constitute gut microbiota and play an essential role in aiding in the digestion and absorption of nutrients and influencing the efficiency of energy harvest from diet (Janssen and Kersten, 2015; Gerard, 2016). Fecal microbiota transplantation (FMT) from obese humans or mice to germ-free (GF) or antibiotic-treated mice resulted in an increase in body fat deposition (Turnbaugh et al., 2006; Ridaura et al., 2013; Ellekilde et al., 2014), suggesting that intestinal microbiota plays an important role in the regulation of fat metabolism (Greiner and Backhed, 2011). Consistently, two recent studies also suggested that FMT could transfer some of the digestive, myogenic and lipogenic characteristics from pigs to germ-free mice (Diao et al., 2016; Yan et al., 2016).

We recently revealed drastic differences in gut bacterial community structure between Jinhua and Landrace pigs (Xiao et al., 2018). Functional prediction of the bacterial community suggested an increased fatty acid biosynthesis in Jinhua pigs (Xiao et al., 2018). To examine whether metabolic characteristics of Jinhua pigs are controlled by gut microbiota and transferrable to naive animals, we transplanted fecal microbiota of Jinhua and Landrace pigs separately to antibiotic-treated mice. Intestinal gut bacterial community structure and fat metabolism were monitored in mouse recipients. We provided unequivocal evidence to show that gut microbiota is a major contributor to the propensity for adiposity in pigs and possibly other animal species as well.

## Materials and Methods

### Ethics Statement

This study was carried out in accordance with the recommendations of Ethical Committee of Zhejiang Academy of Agricultural Sciences. The protocol was approved by the Animal Care and Use Committee of Zhejiang Academy of Agricultural Sciences (ZAAS-2016-012).

### Pigs and Sample Collection

A total of 10 Jinhua and 10 Landrace pigs, with five males and five females of similar weights in each breed, were housed in the same environmentally controlled room in a swine breeding farm and fed a standard commercial corn-soybean diet. At 240 days of age, fresh feces were collected from each animal, mixed in equal amounts according to the breed to generate a “representative” fecal sample with aggregated microbiome of each breed, and then stored at -80°C for future FMT. At day 240, the pigs were also weighed (Xiao et al., 2018) and sacrificed for collection of blood and tissue samples. Segments of the liver, abdominal adipose tissue, and intestinal tract were used for biochemical measurements, RNA isolation, or perfused with OCT (optimal cutting temperature compound; Sakura Finetek, Torrance, CA, United States), cryo-preserved, and sectioned for histological staining as detailed below.

### Fecal Microbiota Transplantation and Sample Collection

A total of 12 male and 12 female C57BL/6J mice at 4 weeks of age were employed as recipients of swine gut microbiota. Mice were maintained aseptically in gnotobiotic isolators in SPF Animal Technology Co. (Beijing, China), under a 12-h light and 12-h dark cycle with free access to autoclaved water and mouse chow. Prior to FMT, an antibiotic cocktail (0.5 g/L vancomycin, 1 g/L neomycin sulfate, 1 g/L metronidazole, and 1 g/L ampicillin) was administered in drinking water *ad libitum* to mice to deplete commensal bacteria as described (Rakoff-Nahoum et al., 2004; Wang et al., 2011). After 28 days of continuous treatment, mice were randomly divided into two groups of 12 with an equal number of males and females in each group for FMT. Swine fecal microbiota suspension was prepared as described (Pang et al., 2007). In brief, the fecal samples were diluted 5-fold (w/v) in sterile phosphate buffered saline (PBS) and homogenized. The suspension was mixed thoroughly and let stand for 1 min. The supernatant was then aliquoted and stored at -80°C. The FMT procedure was performed daily for 7 days. On each FMT day, 0.2 mL of fecal supernatant was administered to each mouse via intragastric gavage. Animals were maintained for another 28 days after the last inoculation. Fresh feces of the mice were collected before fecal inoculation as well as on days 1, 2, 4, and 7 after initial inoculation and then weekly for another 4 weeks. Bacterial DNA was isolated from fecal materials for subsequent denaturing gradient gel electrophoresis (DGGE). All mice were sacrificed on day 28 after the last fecal inoculation, and the cecal contents were obtained for 16S rRNA gene sequencing. Segments of the liver, abdominal adipose tissue, and intestinal tract were collected for RNA isolation and/or cryo-sectioning. The blood, liver, and abdominal adipose tissues were also sampled for biochemical measurements of various metabolites as described below.

### Biochemical and Enzymatic Measurements

Sera of pigs and mice were prepared from the blood samples and used to measure glucose, low-density lipoprotein (LDL) cholesterol, high-density lipoprotein (HDL) cholesterol, triglyceride, albumin, total protein, and urea nitrogen using an Automatic Biochemical Analyzer (Beckman, Miami, FL, United States) according to the manufacturer’s instructions. Approximately 100 mg of frozen liver and abdominal adipose tissue samples were homogenized in 1 ml of ice-cold PBS. The LPL activity and the triglyceride content were determined in tissue homogenates using respective assay kits according to the manufacturer’s instructions (Jiancheng Bioengineering Ltd, Nanjing, China). The LPL activity was expressed as U/mg protein of the liver tissue, while the triglyceride content was expressed as mmol/g protein.

### Quantitative RT-PCR (RT-qPCR)

Total RNA was isolated using RNeasy Plus Mini Kit (Qiagen) and reverse transcribed using SuperScript II Reverse Transcription Kit (Invitrogen) according to the manufacturer’s instructions. Quantitative PCR assays were performed in triplicate on an ABI Prism 7700 Sequence Detector (Applied Biosystems) using an annealing temperature of 63°C and gene-specific primers listed in Supplementary Tables S1, S2. Data were normalized to glyceraldehyde 3-phosphate dehydrogenase (GAPDH) or 18S rRNA using the comparative ΔΔCt method as described (Xiao et al., 2017b).

### Histological Staining

Pig and mouse liver and adipose tissue segments were fixed in 4% paraformaldehyde for 1 h at room temperature, cryoprotected in 20% sucrose at 4°C overnight, and embedded in OCT. A series of 12-μm cryosections were prepared and stained with hematoxylin, eosin, and/or Oil Red O (Sigma-Aldrich, St. Louis, MO, United States).

### Denaturing Gradient Gel Electrophoresis (DGGE)

Microbial genomic DNA was extracted from each fecal sample as previously described (19). The V3-V4 region of bacterial 16S rRNA gene was amplified using primers 341F and 517R. Amplicons were then separated by DGGE in 8% acrylamide gel containing a denaturing gradient (urea and formamide) of 25% to 65% as described (Hufeldt et al., 2010). The gel was then stained with 0.2% AgNO_3_, scanned with a GS-800 Calibrated Densitometer (Bio-Rad) and analyzed with the Molecular Analyst software (version 1.61, Bio-Rad).

### Illumina HiSeq Sequencing and Data Analysis

Next generation sequencing of bacterial genomic DNA and data analysis were performed as we described (Xiao et al., 2018). Briefly, the V4 region of bacterial 16S rRNA gene was amplified from each genomic DNA sample by using the barcode-fusion primers 515F and 806R. Amplicons between 300 and 350 bp were extracted from agarose gel using a GeneJET Gel Extraction Kit (Thermo Scientific) and pooled in equimolar ratios. Sequencing libraries were then constructed using TruSeq DNA PCR-Free Library Preparation Kit (Illumina) and sequenced commercially on an Illumina HiSeq platform by Novogene (Beijing, China), and 250-bp paired-end reads were generated, which were subsequently analyzed using the QIIME software package (Lawley and Tannock, 2017). Raw reads were first filtered for quality before being merged into tags using FLASH (Magoc and Salzberg, 2011), which were then assigned to each sample according to the unique barcodes. Operational taxonomic units (OTUs) were assembled with a 97% identity threshold, and a representative sequence was picked for each OTU and annotated with taxonomic information using the RDP classifier (Wang et al., 2007). Pie charts showing taxa distribution at the phylum and genus levels were constructed. Principal coordinate analysis (PCoA) was conducted to illustrate the β-diversity based on weighted UniFrac distances.

The sequencing data involved in this paper were deposited in NCBI Sequence Read Archive (SRA) associated with BioProject ID PRJNA487066.

### Statistical Analysis

Data are expressed as means ± SEM. All statistical analyses were performed using SPSS (SPSS, Chicago, IL, United States). The differences between two groups were analyzed using unpaired two-tailed Student’s *t*-test. Test results of all statistical analyses were considered significant if *P* < 0.05.

## Results

### Jinhua Pigs Have a Higher Propensity for Lipogenesis

**FIGURE 1:**
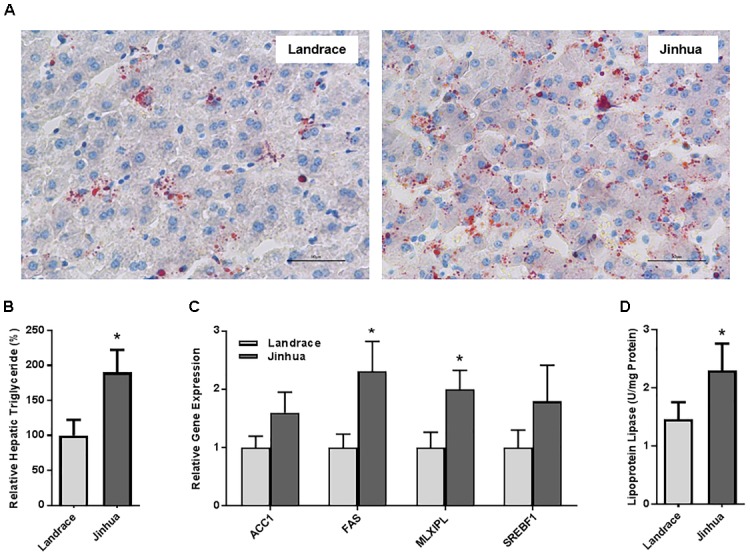
Fat deposition and lipogenesis in the liver of Landrace and Jinhua pigs. **(A)** Representative pictures of the liver sections of Landrace and Jinhua pigs stained with Oil Red O. Neutral lipids appear in red (magnification: 400×). **(B)** Relative triglyceride content (%) in the liver of Landrace and Jinhua pigs. **(C)** Relative expression levels of several major lipogenic genes in the liver of two breeds of pigs. **(D)** The lipoprotein lipase activity in the liver of Landrace and Jinhua pigs. The results were shown as means ± SEM of 10 pigs. ^∗^*P* < 0.05 (by unpaired Student’s *t*-test).

A total of 10 Jinhua and 10 Landrace pigs, with five males and five females in each breed, were housed in an environmentally controlled room with free access to a common standard diet. On day 240, blood, liver, abdominal adipose tissue, and intestinal samples were collected from each pig to examine the differences in fat metabolism characteristics between the two breeds. As shown in Table 1, Jinhua pigs had a significantly higher level of serum triglycerides than Landrace pigs (*P* = 0.021), with no obvious difference in serum cholesterol or glucose levels. The liver triglyceride level was also significantly higher in Jinhua pigs than in Landrace pigs as revealed by both Oil Red O staining (Figure 1A) and a quantitative triglyceride assay kit (Figure 1B). Consistently, Jinhua pigs had higher mRNA levels of the genes for two key lipogenic enzymes, ACC1 and FAS, in the liver than Landrace pigs (Figure 1C). MLXIPL and SREBF1, two lipogenic transcription factors, were also expressed at higher levels in the liver of Jinhua pigs (Figure 1C). Moreover, relative to Landrace pigs, the liver of Jinhua pigs had a significantly elevated LPL activity (*P* < 0.05) (Figure 1D).

**Table 1:** Biochemical parameters in the sera of Landrace and Jinhua pigs.

Biochemical parameter	Landrace	Jinhua	SEM	*P*-value
Triglyceride (mmol/L)	0.52	0.76	0.04	0.021
Glucose (mmol/L)	10.64	11.84	1.56	0.464
Cholesterol (mmol/L)	2.29	2.48	0.21	0.209
HDL cholesterol (mmol/L)	0.97	0.63	0.06	0.040
LDL cholesterol (mmol/L)	1.21	1.33	0.13	0.645
Urea (mmol/L)	5.15	7.61	0.42	0.035
Total protein (g/L)	88.42	73.63	8.73	0.043
Albumin (g/L)	42.83	40.66	3.09	0.599

**FIGURE 2:**
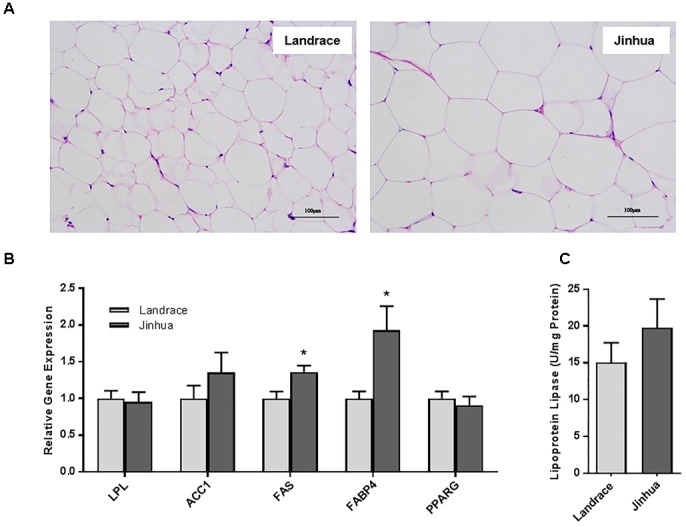
Fat deposition and lipogenesis in the abdominal fat tissue of Landrace and Jinhua pigs. **(A)** Representative pictures of the abdominal fat sections stained with hematoxylin and eosin (magnification: 200×). **(B)** Relative expression levels of several major lipogenic genes in the abdominal fat of two breeds of pigs. **(C)** The lipoprotein lipase activity in the abdominal fat of Landrace and Jinhua pigs. The results were shown as means ± SEM of 10 pigs. ^∗^*P* < 0.05 (by unpaired Student’s *t*-test).

**FIGURE 3:**
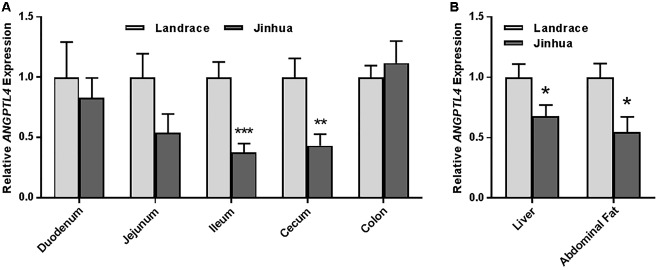
Relative *ANGPTL4* mRNA expression levels in the intestinal tract **(A)** and liver and abdominal fat **(B)** of Landrace and Jinhua pigs. The results were shown as means ± SEM of 10 pigs. ^∗^*P* < 0.05, ^∗∗^*P* < 0.01, and ^∗∗∗^*P* < 0.001 (by unpaired Student’s *t*-test).

Staining of the abdominal adipose tissue showed an increased size of adipocytes in Jinhua pigs as compared to those in Landrace pigs (Figure 2A). Quantitative PCR analysis of the expression levels of the lipogenesis (*ACC1, FAS* and *LPL*) and adipogenesis (*FABP4* and *PPARG*) genes demonstrated an increase in *FAS* and *FABP4* expression in adipocytes of Jinhua pigs, while it was not the case with *ACC1, LPL* or *PPARG* (Figure 2B). The LPL activity in the abdominal fat tissue of Jinhua pigs was numerically higher than that of Landrace pigs, but with no statistical significance (Figure 2C). Relative to Landrace pigs, Jinhua pigs showed generally reduced expression of the *ANGPTL4* mRNA throughout the intestinal tract, with the ileum and cecum being significantly different (*P* < 0.05) (Figure 3A). Similarly, the *ANGPTL4* mRNA was significantly lower in the liver and abdominal fat of Jinhua pigs than those of Landrace pigs (Figure 3B). This is consistent with that fact that Jinhua pigs are more efficient in adipogenesis and fat deposition than Landrace pigs.

### Fecal Microbiota Differs Between Jinhua and Landrace Pigs

**FIGURE 4:**
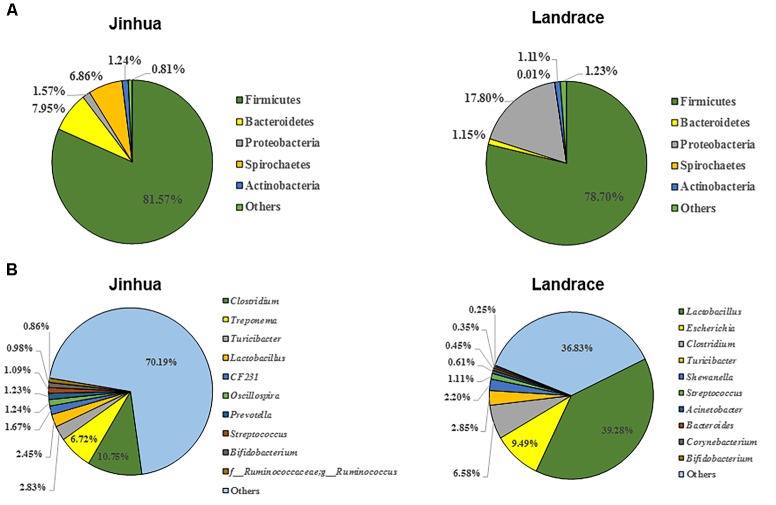
The composition of the fecal bacterial community in Jinhua and Landrace donor pigs at the phylum **(A)** and genus **(B)** levels. Only top 5 phyla and top 10 genera are shown.

As a first step to study the role of gut microbiota in adipogenesis in pigs, we investigated the differences in fecal microbiota between Jinhua and Landrace pigs. Fecal samples from 10 Jinhua and 10 Landrace pigs were collected, pooled by breed, and subjected to bacterial 16S rRNA gene sequencing. A total of 60,064 and 68,014 high-quality sequence tags were generated from the two pooled samples and further classified into 648 and 499 OTUs at the 97% identity level, respectively. The fecal bacterial OTUs were classified using the RDP classifier. The top five major phyla are illustrated in Figure 4A. Firmicutes was the most abundant phylum and accounted for more than 75% of the total sequences in both samples. Bacteroidetes was the second most abundant in Jinhua pigs followed by Spirochaetes, while these two phyla were dramatically diminished in Landrace pigs. On the other hand, Proteobacteria accounted for nearly 18% of all fecal bacteria in Landrace pigs, but was less than 2% in Jinhua pigs (Figure 4A). At the genus level, the top four genera in Jinhua pigs were *Clostridium, Treponema, Turicibacter*, and *Lactobacillus*, while they were *Lactobacillus, Escherichia, Clostridium*, and *Turicibacter* in Landrace pigs (Figure 4B). We also measured the short-chain fatty acid (SCFA) profiles in the cecum and colon of Jinhua and Landrace pigs. It appeared that elevated levels of SCFAs were associated with Landrace pigs (Supplementary Figure S1). Although enhanced SCFA synthesis is associated with improved energy extraction from diet, accumulating evidence suggests a paradoxical role of SCFAs in preventing and counteracting obesity (Canfora et al., 2015), which is consistent with the lean phenotype of Landrace pigs.

### FMT of Pigs Induces Distinct Alterations in Gut Microbiota of Mouse Recipients

To examine whether gut microbiota between the two breeds of pigs could confer phenotypic differences in adiposity, the pigs’ fecal microbiota was transplanted into two groups of 12 mice that had been treated with a cocktail of antibiotics for 28 days. An obvious reduction in the abundance and diversity of fecal bacteria was observed in antibiotic-treated mice gut prior to FMT as revealed by DGGE (Supplementary Figure S2), consistent with previous studies (Rakoff-Nahoum et al., 2004; Wang et al., 2011). Moreover, no obvious difference in fecal microbiota was seen between the two recipient groups prior to FMT. However, daily inoculation of porcine fecal bacteria for 7 days gradually enhanced gut bacterial diversity in mice, which became stabilized 21 or 28 days after the last inoculation (Supplementary Figure S1). All mouse recipients were therefore sacrificed on day 28.

**FIGURE 5:**
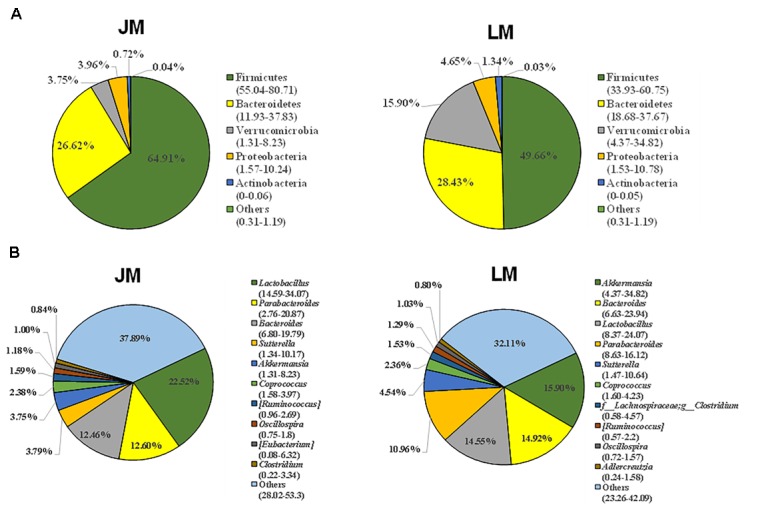
Cecal bacterial community structure of mouse recipients at the phylum **(A)** and genus **(B)** levels. Only top 5 phyla and top 10 genera are shown. JM, mice receiving fecal microbiota from Jinhua pigs; LM, mice receiving fecal microbiota from Landrace pigs.

**FIGURE 6:**
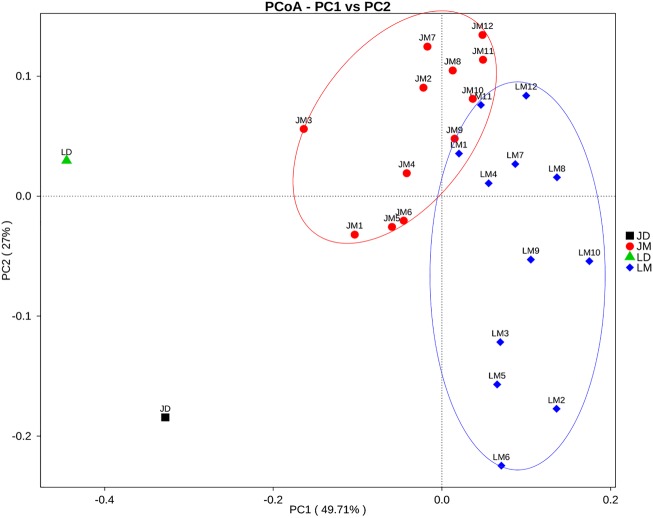
Principal coordinates analysis (PCoA) of the cecal bacterial community composition of mouse recipients based on weighted unifrac distance. JD, Jinhua pig donor; LD, Landrace pig donor; JM, mice receiving fecal microbiota from Jinhua pigs; LM, mice receiving fecal microbiota from Landrace pigs.

The cecal contents were obtained from individual mice and subjected to 16S rRNA gene sequencing for further analysis of the differences in gut bacterial community structure of mouse recipients. A total of 1,128,463 DNA sequence tags were generated from two groups of mice, with an average of 47,019 tags per sample (range = 41,620 ∼ 53,520, SEM = 766.6). The tags were further grouped into 9,602 OTUs at the 97% identity level, with an average OTU number of approximately 400 per sample (range = 264 ∼ 547, SEM = 12.32). Taxonomic analysis indicated that Firmicutes and Bacteroidetes were the two most abundant phyla (Figure 5A). While Bacteroidetes was relatively similar in abundance in both groups of mice, Firmicutes in mice receiving Jinhua pigs’ feces (JM) was approximately 15% more prevalent than those in mice receiving Landrace pigs’ feces (LM), resulting in an elevated ratio of Firmicutes to Bacteroidetes among JM mice, which was associated with the obesity phenotype (Turnbaugh et al., 2008). Notably, *Verrucomicrobia* was greatly reduced in JM mice as compared to LM mice. At the genus level, *Lactobacillus, Akkermansia, Bacteroides, Parabacteroides*, and *Suttertlla* constituted the top five genera in both groups of mice (Figure 5B). Among them, *Akkermansia*, a representative member of the phylum Verrucomicrobia, was remarkably low in JM mice (3.75%) as compared with LM mice (15.9%). *Bacteroides* was also slightly less abundant in LM mice than JM mice (Figure 5B). Consistently, a PCoA plot based on weighted UniFrac revealed a clear segregation between the JM and LM cecal samples (Figure 6), suggesting that transplantation of Landrace and Jinhua fecal microbiota induced distinct alterations in gut microbiota of mouse recipients.

### Mouse Recipients Resemble Their Respective Pig Donors in Fat Metabolism

**FIGURE 7:**
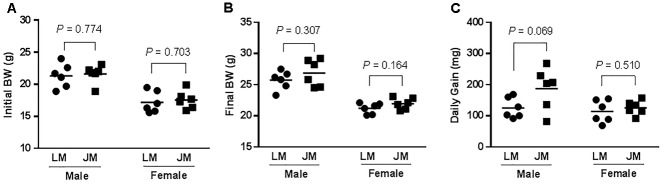
Initial **(A)** and final body weight (BW) **(B)** and daily weight gain **(C)** of mice receiving fecal microbiota of Landrace and Jinhua pigs. The horizontal line in each group denotes the average value of six mice. JM, mice receiving fecal microbiota from Jinhua pigs; LM, mice receiving fecal microbiota from Landrace pigs. Statistical analysis was conducted using unpaired Student’s *t*-test and the significance values were indicated.

To further reveal the impact of FMT of Jinhua and Landrace pigs on fat metabolism in mouse recipients, body weight was recorded 28 days after the last gavage, and the blood, liver, abdominal adipose tissue, and intestinal segments were also collected from mouse recipients. When compared to Landrace pig microbiota, fecal microbiota of Jinhua pigs had a strong tendency to increase daily weight gain of males (*P* = 0.069), but with a less dramatic effect on female recipients (Figure 7). Biochemical analysis of the serum samples of mouse recipients revealed a metabolic profile similar to that of their donors (Table 2). For example, JM mice had a significantly higher serum triglyceride level than LM mice (*P* = 0.050) (Table 2), similar to the difference in their respective donors (Table 1).

**Table 2:** Biochemical parameters in the sera of mice receiving fecal microbiota of Landrace and Jinhua pigs.

Biochemical parameter	LM^a^	JM^a^	SEM	*P*-value
Triglyceride (mmol/L)	1.18	1.68	0.11	0.050
Glucose (mmol/L)	8.73	8.41	0.67	0.578
Cholesterol (mmol/L)	3.99	4.18	0.36	0.746
HDL cholesterol (mmol/L)	4.06	3.23	0.32	0.087
LDL cholesterol (mmol/L)	2.16	2.78	0.21	0.094
Urea (mmol/L)	6.64	8.32	0.50	0.057
Total protein (g/L)	80.53	79.44	8.91	0.901
Albumin (g/L)	47.24	48.86	5.01	0.864

*^*a*^LM, mice receiving fecal microbiota from Landrace pigs; JM, mice receiving fecal microbiota from Jinhua pigs*.

**FIGURE 8:**
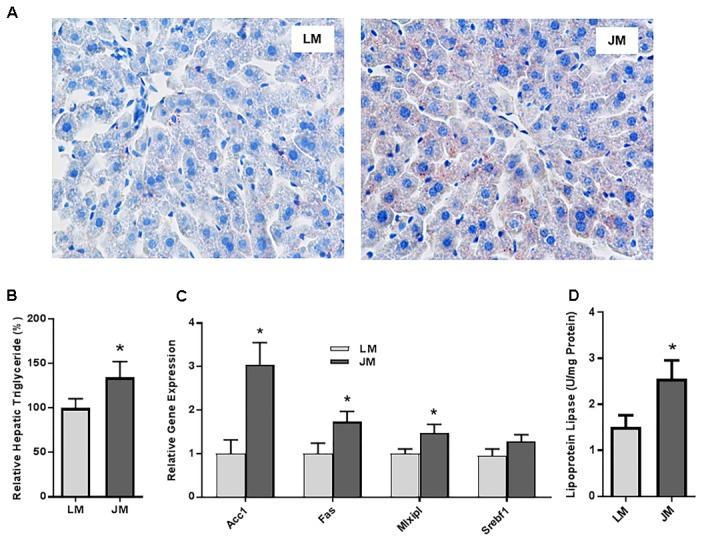
Fat deposition and lipogenesis in the liver of mice transplanted with Landrace and Jinhua pig microbiota. **(A)** Representative pictures of the liver sections of mouse recipients stained with Oil Red O. Neutral lipids appear in red (magnification: 400×). **(B)** Relative triglyceride content (%) in the liver of mouse recipients. **(C)** Relative expression levels of several major lipogenic genes in the liver of mouse recipients. **(D)** The lipoprotein lipase activity in the liver of mouse recipients. The results were shown as means ± SEM of 12 mice. ^∗^*P* < 0.05 (by unpaired Student’s *t*-test). LM, mice receiving fecal microbiota from Landrace pigs; JM, mice receiving fecal microbiota from Jinhua pigs.

**FIGURE 9:**
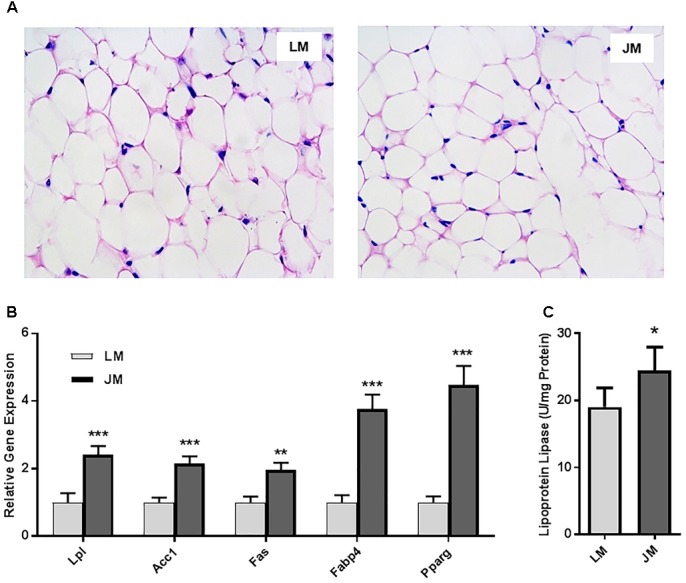
Fat deposition and lipogenesis in the abdominal fat tissue of mouse recipients. **(A)** Representative pictures of the abdominal fat sections stained with hematoxylin and eosin (magnification: 200×). **(B)** Relative expression levels of several major lipogenic genes in the abdominal fat of mice transplanted with Landrace and Jinhua pig microbiota. **(C)** The lipoprotein lipase activity in the abdominal fat of mouse recipients. The results were shown as means ± SEM of 12 mice. ^∗^*P* < 0.05, ^∗∗^*P* < 0.01, and ^∗∗∗^*P* < 0.001 (by unpaired Student’s *t*-test). LM, mice receiving fecal microbiota from Landrace pigs; JM, mice receiving fecal microbiota from Jinhua pigs.

**FIGURE 10:**
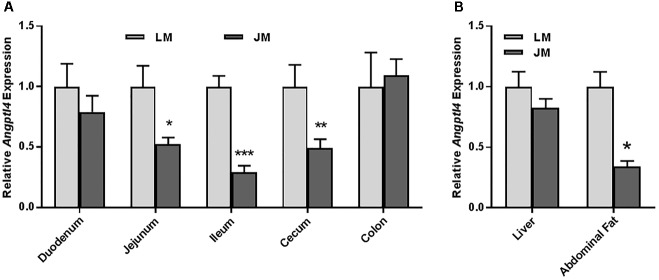
Relative *Angptl4* mRNA expression levels in the intestinal tract **(A)** and the liver and abdominal fat **(B)** of mouse recipients. The results were shown as means ± SEM of 12 mice. ^∗^*P* < 0.05, ^∗∗^*P* < 0.01, and ^∗∗∗^*P* < 0.001 (by unpaired Student’s *t*-test). LM, mice receiving fecal microbiota from Landrace pigs; JM, mice receiving fecal microbiota from Jinhua pigs.

Relative to LM mice, JM mice also showed an increased lipid content in the liver as revealed by the Oil Red O staining (Figure 8A). Consistently, hepatic triglyceride level was significantly increased in JM mice (Figure 8B). The mRNA levels of two lipogenic enzymes (ACC1 and FAS) as well as two lipogenic transcription factors (MLXIPL and SREBF1) were significantly higher or tended to be higher in JM mice (Figure 8C). The LPL activity was also significantly higher in the livers of JM mice than those of LM mice (Figure 8D). Although no evident difference in adipocyte morphology or fat deposition was observed in the abdominal adipose tissue between the two groups of mouse recipients following FMT (Figure 9A), the mRNA levels of *Lpl, Acc1, Fas, Fabp4*, and *Pparg* were markedly upregulated in JM mice than those in LM mice (Figure 9B). Similarly, the LPL activity was significantly higher in the adipose tissue of JM mice than LM mice (Figure 9C). Relative to LM mice, JM mice also showed a significant reduction in the *Angptl4* mRNA expression level in the jejunum, ileum, cecum, and abdominal fat tissue (Figures 10A,B). Collectively, these observations strongly indicated that the mouse recipients exhibited similar characteristics in fat metabolism as their respective pig donors, suggesting that gut microbiota of obese Jinhua pigs is capable of enhancing lipogenesis with an increased capacity to harvest energy from the diet than that of lean Landrace, and that this capacity is transferrable across species.

## Discussion

Jinhua and Landrace pigs display notable differences in the body fat content and propensity for adipogenesis and represent good models to study human overweight and obesity (Miao et al., 2009; Guo et al., 2011). Recent studies indicated that gut microbiota plays a critical role in energy harvest from the diet and subsequent fat metabolism and deposition (Turnbaugh et al., 2006; Greiner and Backhed, 2011; Gerard, 2016). In addition to facilitating the hydrolysis of indigestible dietary fibers to absorbable monosaccharides, gut microbiota could potentially upregulate lipogenic enzymes, ACC1 and FAS, via transcriptional factors such as MLXIPL and SREBF1 and suppress ANGPTL4, a circulating LPL inhibitor, resulting in an increased LPL activity (Strable and Ntambi, 2010). Consistent with the differences in fat metabolism between the two breeds of pigs, we indeed observed enhanced mRNA levels of most key lipogenic genes and reduced *ANGPTL4* expression in Jinhua pigs at varying degrees compared with those in Landrace pigs. Moreover, the LPL activities in the liver and adipose tissue of Jinhua pigs were also higher than those in Landrace pigs.

Direct bacterial 16S rRNA gene amplicon sequencing of fecal microbiota in the two pig breeds revealed a remarkable difference in microbial community structure. Except for Firmicutes, which dominated fecal microbiota in both breeds, most of other phyla such as Bacteroidetes and Proteobacteria evidently differed in relative abundance, with the former accounting for a bigger fraction in Jinhua pigs and the latter being more abundant in Landrace pigs. Members of Proteobacteria are known to be associated with different diseases and an increase in the relative abundance of Proteobacteria has been considered as a sign of dysbiosis (Shin et al., 2015). However, in contrast with previous studies (Shin et al., 2015), where Proteobacteria was associated with obesity, our data showed that the Landrace pig, which has the lean phenotype, was enriched with Proteobacteria. Interestingly, this phylum did not differ among the mice receiving Jinghua or Landrace microbiota, suggesting that Proteobacteria might not play an important role in fat metabolism. Nevertheless, because Proteobacteria consists of a large number of different bacterial species, caution should be taken when drawing any conclusion on the involvement of this phylum in lipogenesis. The most notable difference in the bacterial relative abundance at the genus level was the probiotic bacterial genus, *Lactobacillus*, which was significantly more abundant in Landrace pigs than in Jinhua pigs. In line with a higher *ANGPTL4* expression in Landrace pigs, a *Lactobacillus* species, *L. paracasei* subsp. *paracasei* F19 was recently shown to upregulate the *ANGPTL4* mRNA expression and reduce body fat composition (Aronsson et al., 2010). Differential expression of many other gut bacteria obviously contributed to the overall lipogenic phenotype in pigs.

To evaluate relative contributions of gut microbiota to phenotypic differences in fat metabolism between the two breeds of pigs and to eliminate the impact of genetics, we further transferred the fecal microbiota of Jinhua and Landrace pigs separately to antibiotic-treated C57BL/6J mice. Markedly different structures of the gut bacterial community were found in mice following FMT. We observed an elevated ratio of Firmicutes to Bacteroidetes in JM mice, which is also associated with obese human populations (Bervoets et al., 2013). A significant diminishment of *Akkermansia*, a mucin-degrading bacterium residing in the mucus layer, was also seen in JM mice. Perhaps not coincidently, *Akkermansia* is known to be inversely correlated with obesity (Cani and de Vos, 2017). *A. muciniphila* produces SCFAs, activates fatty acid oxidation, and inhibits fatty acid synthesis, resulting in a reduction in the accumulation of free fatty acids in circulation and a decrease in body weight (Besten et al., 2013; Derrien et al., 2017). In fact, *A. muciniphila* is beneficial in reversing high-fat diet-induced metabolic disorders such as obesity and insulin resistance (Everard et al., 2013).

Importantly, our results clearly indicated that gut microbiota recapitulated most of the donor phenotypes in respective mouse recipients. For example, JM mice inoculated with Jinhua pig’s feces showed elevated lipid and triglyceride contents and the LPL activity in the liver as compared to LM mice inoculated with Landrace pig’s feces. At the same time, the expression levels of the genes for several key lipogenic enzymes and transcription factors were increased, while the *Angptl4* expression was reduced in mice following fecal transplantation of Jinhua pigs. In agreement with our observations, transplantation of human microbiota has been found to modify energy metabolism and increase body and fat mass in germ-free mice (Ridaura et al., 2013). Similarly, two recent studies also revealed the FMT could transfer many of the host characteristics such as myogenesis, lipogenesis, and the antioxidant and digestive enzyme activities from pigs to germ-free mice (Diao et al., 2016; Yan et al., 2016). This study revealed the feasibility and reliability of cross-species transmission of the donor phenotypes to antibiotic-treated, rather than germ-free, mice, opening the door to convenient evaluations of the role of non-rodent gut microbiome under different pathophysiological conditions in rodent models.

Consistent with increased expressions of lipogenic genes and fat deposition in the liver as well as elevated blood lipid profiles in JM mice following FMT, we observed a strong tendency for enhanced weight gain. Such gain is very likely due to augmented fat deposition and an increase in the fat content as seen in Jinhua donor pigs. This study has opened possibilities for manipulating the growth performance, fat content, and meat quality of lean commercial pig breeds through large-scale FMT of obese pig microbiota or by co-mingling obese with lean pig breeds. However, the influence of donor’s “obese” or “lean” microbiota on food intake is unknown, but likely minimum, because no obvious difference was observed in food intake between JM and LM mice, although mouse chow was provided *ad libitum*. Obesity has been linked to chronic inflammation (Boulangé et al., 2016) and obese pigs show increased inflammation systemically in the adipose tissue (Renner et al., 2018). It will be interesting to evaluate whether Jinhua pigs and JM mice harboring “obese” microbiota exhibit heightened intestinal and systemic inflammation and whether they are more prone to produce inflammatory mediators when triggered.

It is noted that ANGPTL4 is primarily involved in lipid metabolism (Backhed et al., 2004; Dijk and Kersten, 2016; Olshan and Rader, 2018), but an inactivation of *ANGPTL4* was recently shown to improve glucose tolerance (Gusarova et al., 2018; Janssen et al., 2018; Singh et al., 2018). In this study, we observed largely a decrease in the expression of *ANGPTL4* in the intestine, liver and abdominal fat of Jinhua pigs and JM mice, which is consistent with enhanced lipogenic potential of the animals. However, we failed to see any obvious difference in the blood glucose level between Jinhua and Landrace pigs and between JM and LM mice, suggestive of a limited impact of different microbiota on glucose metabolism.

Uremia is a metabolic disorder denoting a very high urea concentration in the blood that is the result of renal or liver dysfunction (Vitetta and Gobe, 2013). Jinhua pigs and JM mice show a strong tendency with increased blood urea levels relative to their counterparts. It is, however, very unlikely to amount to the level of uremia, because both Jinhua pigs and JM mice behave and mature normally with no clinical signs of uremia such as loss of appetite, muscle atrophy, fatigue, and tremors. In fact, the serum urea levels of healthy Landrace pigs are in the range of 3.0–6.8 mM (Elbers et al., 1994). In this study, we observed an average 5.15 mM in Landrace pigs, while Jinhua pigs had a serum urea concentration of 7.61 mM (Table 1), which is only slightly above the upper limit of Landrace pigs. An increase in blood urea in Jinhua pigs and JM mice is likely due to an enhanced ability of their gut microbiota in protein and amino acid metabolism, resulting in the production of an excessive amount of metabolic products such as urea and creatinine.

The 4-week antibiotic regimen involving the use of a combination of ampicillin, vancomycin, neomycin, and metronidazole in drinking water was adopted from an earlier study (Rakoff-Nahoum et al., 2004). A depletion of all culturable, aerobic and anaerobic commensal bacteria was reported in the colonic content of mice following the antibiotic treatment (Rakoff-Nahoum et al., 2004), which is consistent with a substantial decrease in the diversity and abundance of gut bacteria that we observed in the DGGE profile. Although residual commensal bacteria might impact the FMT outcomes, we observed a recapitulation of pig donors’ phenotypes in fat metabolism in the respective groups of mouse recipients following FMT. Nevertheless, it is more desirable to use germ-free mice in FMT to completely eliminate the potential complications of the recipient’s commercial bacteria.

In summary, transplantation of lean and obese pig microbiota has led to recapitulation of the donor’s phenotypes in respective mouse recipients, reinforcing the notion that gut microbiome plays a critical role in host metabolic health. This study has convincingly demonstrated that: (1) the obese gut microbiota of Jinhua pigs has the tendency to facilitate fat deposition and contributes to body composition and meat quality, (2) cross-species transmission of the adiposity phenotype is feasible, and (3) treating mice with broad-spectrum antibiotics is a convenient alternative approach to the germ-free technology for cross-species transplantation of gut microbiota. Therefore, we have provided evidence to link the differences in gut microbiota community structures of Jinhua and Landrace pigs to their respective fat metabolism phenotypes, suggesting the potential for manipulating gut microbiota to improve growth performance and meat quality of lean commercial pig breeds. In addition, given their similarities in anatomy and size, pigs are a valuable model for human obesity studies.

## Author Contributions

HY, YX, and YpX conceived and designed the experiments. HY, YX, JW, and YpX performed the experiments. HY, KR, JW, YpX, JZ, and GZ analyzed and interpreted the data. HY, KR, YpX, JZ, and GZ drafted and revised the manuscript.

## Conflict of Interest Statement

The authors declare that the research was conducted in the absence of any commercial or financial relationships that could be construed as a potential conflict of interest.
