# Supplementary material for: Gut Microbiota Is a Major Contributor to Adiposity in Pigs
Source: Front Microbiol. 2018 Dec 10;9:3045. doi: 10.3389/fmicb.2018.03045 (PMC6296290; doi:10.3389/fmicb.2018.03045)
Supplement: Supplementary file 1 [file Data_Sheet_1.docx]

**Gut Microbiota Is a Major Contributor to Adiposity in Pigs**

**Hua Yang^1^, Yun Xiang^2^, Kelsy Robinson^3^, Junjun Wang^4^, Guolong Zhang^3^*, Jiangchao Zhao^5^*, Yingping Xiao^1^***

^1^ Institute of Quality and Standards for Agro-products, Zhejiang Academy of Agricultural Sciences, Hangzhou, Zhejiang, China

^2^ Institute of Animal Husbandry and Veterinary Medicine, Jinhua Academy of Agricultural Sciences, Jinhua, Zhejiang, China

^3^ Department of Animal and Food Sciences, Oklahoma State University, Stillwater, Oklahoma, USA

^4^ Beijing Advanced Innovation Center for Food Nutrition and Human Health; State Key Laboratory of Animal Nutrition, China Agricultural University, Beijing, China

^5^ Department of Animal Science, Division of Agriculture, University of Arkansas, Fayetteville, Arkansas, USA

*** Correspondence:** zguolon@okstate.edu; jzhao77@uark.edu; ypxiaozj@hotmail.com

**Table S1. Real-Time PCR Primers for Porcine Genes**

| Gene | GenBank Accession No. | Primer Sequences (5' to 3') | Size (bp) |
| --- | --- | --- | --- |
| *ANGPTL4* | NM_001038644 | CGACCTCCGAGGAGACAAGAA | 108 |
|  |  | CGAGGGATGGAATGGAAGTACTG |  |
| *GAPDH* | AF017079 | GGCAAATTCCACGGCACAGTCA | 82 |
|  |  | CTCGCTCCTGGAAGATGGTGAT |  |
| *18S* | NR_046261 | GCCCTATCAACTTTCGATGGTAGTC | 113 |
|  |  | CCTTGGATGTGGTAGCCGTTTCTCA |  |
| *ACC1* | NM_001114269 | GGAGGAATACCCGTGGGAGTAGT | 105 |
|  |  | CTGCTGGATTATCTTGGCTTCAGA |  |
| *FAS* | NM_001099930 | CCTCCCTCAACTTCCGAGACG | 152 |
|  |  | CGCGGGCACCATTCCCATCA |  |
| *MLXIPL* | XM_003124408 | GTCCGACATCTCCGACACACTCT | 97 |
|  |  | CATGTCAGCATTGCCGACATAG |  |
| *SREBF1* | NM_214157 | GCACTTTCTGACCCGCTTCTTC | 82 |
|  |  | CTGCATGGCAACAGGCACCGA |  |
| *LPL* | NM_214286 | CCCTATACAAGAGGGAACCGGAT | 138 |
|  |  | CCGCCATCCAGTCGATAAACGT |  |
| *FABP4* | NM_001002817 | CCCAACCTGATCATCACTGTGAAT | 89 |
|  |  | CCCAATTTGAAGGCAATCTCAGT |  |
| *PPARG* | NM_214379 | GTGGAGACCGCCCAGGTTTG | 108 |
|  |  | GGGAGGACTCTGGGTGGTTCA |  |

**Table S2. Real-Time PCR Primers for Mouse Genes**

| Gene | GenBank Accession No. | Primer Sequences (5' to 3') | Size (bp) |
| --- | --- | --- | --- |
| *Angptl4* | NM_020581 | CCTACAAGGATGGCTTCGGAGAT | 86 |
|  |  | GCTTCCTCGGTTCCCTGTGAT |  |
| *Gapdh* | GU214026 | CAGTATGACTCCACTCACGGCAA | 100 |
|  |  | CTCGCTCCTGGAAGATGGTGAT |  |
| *18S* | NR_003278 | CGGACACGGACAGGATTGACA | 94 |
|  |  | CCAGACAAATCGCTCCACCAACTA |  |
| *Fas* | NM_007988 | GCTTTGCTGCCGTGTCCTTCTA | 102 |
|  |  | CTGTCTTGGCACGCAGCAGT |  |
| *Acc1* | NM_133360 | GATGGCGTCCGCTCTGTGATA | 140 |
|  |  | GGTGAGATGTGCTGGGTCATGT |  |
| *Mlxipl* | NM_021455 | GAGCTCAACGCTGCCATCAAC | 93 |
|  |  | CATGTCCCGCATCTGGTCAAA |  |
| *Srebf1* | NM_011480 | CCCGGCTATTCCGTGAACAT | 111 |
|  |  | GCAGATATCCAAGGGCATCTGA |  |
| *Lpl* | NM_008509 | CCAAGCTGGTGGGAAATGATGTG | 95 |
|  |  | GCTGTACCCTAAGAGGTGGACGTT |  |
| *Fabp4* | NM_024406 | GATCATCAGCGTAAATGGGGATT | 118 |
|  |  | CTTCCTGTCGTCTGCGGTGAT |  |
| *Pparg* | NM_001127330 | CCAAGAATACCAAAGTGCGATCA | 133 |
|  |  | CCCACAGACTCGGCACTCAAT |  |


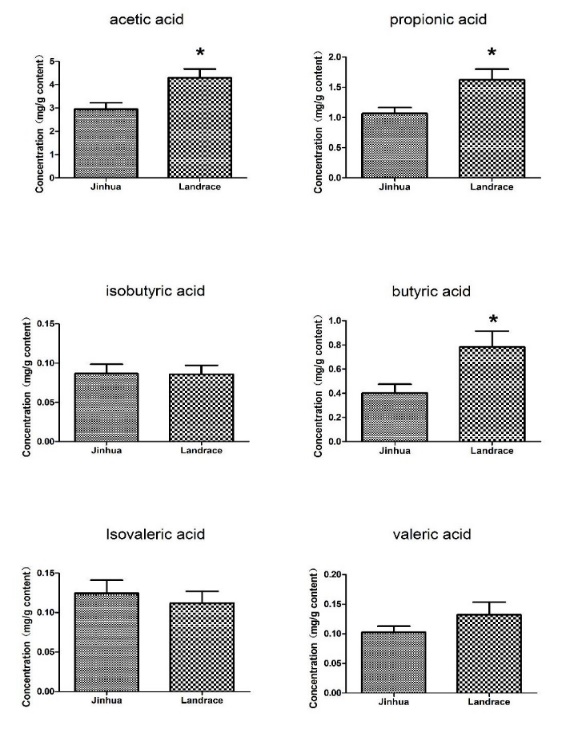

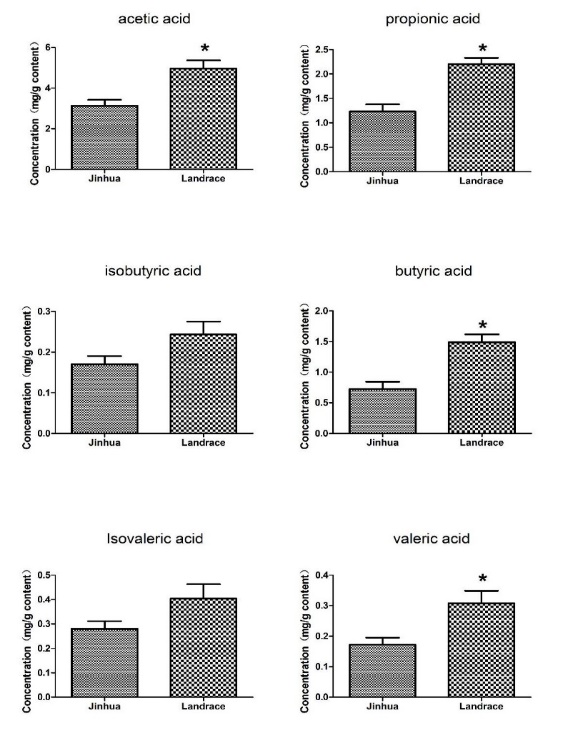


A

B

**Figure S1. Concentrations of short-chain fatty acids in the cecal (A) and colonic contents of 240-day-old Jinhua and Landrace pigs (n = 10).** **P* < 0.05 (by unpaired Student’s *t*-test).

**Fig. S2. Kinetics of the DGGE bacterial profiles of fecal samples from pig donors and mouse recipients before and after fecal microbiota transplantation.** Fecal transplantation was performed daily for 7 days and fecal swaps were taken from mice at various times during and after fecal gavage. Fecal bacterial DNA from pig donors and mouse recipients were analyzed by DGGE. JM, mice receiving fecal microbiota from Jinhua pigs; LM, mice receiving fecal microbiota from Landrace pigs; M, DNA ladder; 0, mice before fecal transplantation; JD, Jinhua pig donors; LD, Landrace pig donors. Each lane represents the fecal microbiota of 12 pooled feces within one group.
